# The Opium Habit

**Published:** 1882-10

**Authors:** William Murrell


### THE OPIUM HABIT, ITS SUCCESSFUL TREATMENT BY THE AVENA SATIVA (?)

The remedy to which Dr. Sell ascribes such remarkable properties (*New York Med. Gaz.*, April 22d) is a tincture of common oats. He begins by telling us that it is a very important grain, and then gives us a long history of its cultivation and uses from the time of Pliny to the present day. He discourses, too, at some length on the value of water-gruel and oatmeal-tea. He next tells us that in 1874 Dr. Keith had a concentrated tincture of the avena prepared for parlysis, from the effects of which he himself suffered for three years and a half, and in three weeks, having taken the avena in fifteen-drop doses three or four times a day, he was not only free from paralysis, but relieved from many serious symptoms, both mental and physical' (!) The author commenced his own observations with the concentrated tincture before hearing Dr. Keith's case. He is convinced that it is 'a most useful and reliable remedy.' He finds that it is 'diuretic, slightly laxative. tonic, stimulant, but especially nerve-stimulant.' It is said to 'exert a most powerful influence upon and through the nervous system.' It is 'a valuable adjuvant to other medicines,' is unsurpassed in 'female diseases,' is 'an excellent substitute for intoxicating drinks, ' 'will cure inebrity,' is 'an antidote to opium-poisoning' (!) It gives relief, in insomnia, and is curative in nervous headache and prostration due to mental strain and worry and also in neuralgia and hemiplega. 'Epilepsy has been brought under subjection by it more effectively than by other remedies,' and it is unequalled in the treatment of 'hysteria, melancholia, neurasthenia, and all forms of nervous prostration, whether caused by inebriety, the abuse of tobacco, opium, or morphine,by sexual excesses, masturbation, or mental strain.' But this is not all, for the author has made what he describes as 'a no small discovery in therapeutics.' He finds that this remedy is an absolute cure for the opium or morphia habit. He gives details of three cases of morphia-taking, all of which were promptly cured by his remedy. The first patient, a German, of middle age, usually took hypodermically in the twenty-four hours from 12 to 48 or 50 grains of morphia, which had no other effect than to produce fifteen minute sleep with the eyes wide open. The next patient, a middle-aged lady, had been a slave of the morphia habit for seven years, and usually took 12 grains a day. In the third case morphia had been taken to excess for twenty years, the average being an ounce in fifteen days, or thirty-two grains a day. The avena in all cases works wonders. The patients are not only able to relinquish the habit, but improve in weight, strength, spirits and mental capacity. One lady not only gained twenty-five pounds in weight in an incredibly short time, but said she felt twenty years younger. It is stated in the most positive terms that the preparation is nothing but a tincture of common oats. The dose is to be increased till it produces 'the desired effect,'and is to be given in hot water, 'with the same frequency that the patient was accustomed to take his opium or morphia.' The author displays great originality, and his powers of imagination are remarkable.—William Murrell, M. D., in *The London Medical Record*, 1882.

